# Association between glucose-to-lymphocyte ratio and mortality in patients with heart failure from the MIMIC-IV database: a retrospective cohort study

**DOI:** 10.1038/s41598-025-08349-9

**Published:** 2025-07-01

**Authors:** Gang Wu, Huanya Ke, Zijia Tong, Jie Yang, Juan Yang, Zhengjun Shen

**Affiliations:** 1 Department of Cardiology, Huanggang Central Hospital, Huanggang, China; 2 Department of Pharmacy, Huanggang Central Hospital, Huanggang, China

**Keywords:** Glucose-to-lymphocyte ratio, GLR, Heart failure, Mortality, MIMIC-IV, Prognostic predictor, Heart failure, Diabetes complications, Heart failure, Risk factors, Prognostic markers

## Abstract

The glucose-to-lymphocyte ratio (GLR) has been associated with prognosis in various inflammatory diseases. However, its relationship with mortality among critically ill patients with heart failure admitted to the intensive care unit (ICU) remains poorly understood. This study aims to assess the association between GLR levels and mortality in this population and to evaluate the prognostic predictive value of GLR. This retrospective cohort study utilized data from the Medical Information Mart for Intensive Care IV (MIMIC-IV3.0) database, encompassing heart failure patients admitted between 2008 and 2022. The GLR was calculated as fasting glucose (mg/dL) divided by absolute lymphocyte count (K/µL). To evaluate the association between GLR levels and 30-day and 365-day mortality risk in heart failure patients, we employed RCS analysis, multivariable Cox regression, K-M survival curves, subgroup analyses, and ROC curves. These methods were used to assess both the prognostic relationship and the predictive accuracy of GLR. This study included a total of 14,417 patients. The association between GLR levels and all-cause mortality was found to be non-linear. In our analysis, GLR was found to be an independent predictor of both 30-day (HR 1.57, 95% CI 1.45–1.70) and 365-day mortality (HR 1.48, 95% CI 1.40–1.56). K-M survival curve analyzes showed that patients with elevated GLR levels had worse survival outcomes than patients with lower levels. Furthermore, the predictive utility of GLR appeared to exceed that of glucose or lymphocyte counts alone. GLR may be a useful tool for early identification and treatment of high-risk populations in clinical practice and may also be a potential predictor of the prognosis of patients with heart failure.

**Supplementary Information:**

The online version contains supplementary material available at 10.1038/s41598-025-08349-9.

## Introduction

Heart failure is a syndrome caused by a number of heart diseases that reach an advanced stage. Heart failure affects approximately 26 million people worldwide, with an average of 3.6 million new cases diagnosed each year. This condition affects 5.7 million people in the United States and is a leading cause of hospitalization^1^. The INTER-CHF prospective cohort study found that the global death rate from heart failure is 16.5%, with the highest rate recorded at 34% in Africa^1,2^. In addition, the annual medical costs associated with heart failure are estimated at approximately $108 billion, representing a significant economic burden. This number is expected to increase due to the world’s aging population, rapid expansion and industrialization^3^. In this context, heart failure has emerged as a serious public health problem characterized by high incidence, poor prognosis, high mortality, and significant medical costs. Therefore, identifying markers that may help assess the prognosis of heart failure and determining appropriate treatment approaches is crucial.

Numerous studies have shown that a sustained inflammatory response contributes to adverse cardiac remodeling and dysfunction, potentially leading to heart failure^4^. For example, Xiaojing et al. have shown that lymphocytes play a critical role in the inflammatory response associated with heart failure^5^. Furthermore, reduced lymphocyte counts are associated with an increased risk of mortality in patients with heart failure^6^. Moreover, lymphocyte subpopulations have been implicated in the pathogenesis of various fibrotic diseases, and in the context of T cell-mediated inflammation, they are particularly relevant to the mechanisms underlying cardiac fibrosis^7^. On the other hand, heart failure is characterized by impaired energy metabolism, and energy deficiency further worsens the severity of heart failure^8^. As a direct source of energy, the uptake and utilization of glucose are crucial for the progression of heart failure^9^. Importantly, dysregulated glucose metabolism, manifested as hyperglycemia, exerts detrimental effects on the cardiovascular system through multiple pathways. Chronically elevated blood glucose levels (e.g., as seen in diabetes mellitus) are strongly associated with the development and progression of heart failure. Persistent hyperglycemia promotes capillary damage, myocardial hypertrophy, and myocardial fibrosis, directly impairing cardiac function^10^. Mechanistically, chronic hyperglycemia drives the formation of advanced glycation end products (AGEs), activates protein kinase C (PKC) isoforms, and increases flux through the hexosamine pathway, collectively contributing to oxidative stress, inflammation, endothelial dysfunction, and extracellular matrix remodeling in the heart^10,11^. This chronic metabolic disturbance significantly increases the risk of developing heart failure and worsens outcomes in existing heart failure^12^.

Acutely elevated blood glucose levels, such as those occurring during acute myocardial infarction or decompensated heart failure episodes (often termed “stress hyperglycemia”), also have profound negative consequences. Even transient hyperglycemia can impair endothelial function, promote thrombosis, increase inflammation, and exacerbate ischemia-reperfusion injury^13,14^. In the context of acute cardiac events, higher admission glucose levels are consistently associated with larger infarct sizes, worse left ventricular function, and increased short- and long-term mortality, independent of diabetic status^15,16^. The detrimental impact of acute hyperglycemia appears to be dose-dependent, with higher levels conferring greater risk^15^.

Moreover, both chronic and acute hyperglycemia inhibit antioxidant signaling pathways, activates inflammatory signaling, and increases the production of reactive oxygen species (ROS) and inflammatory mediators, thereby promoting and exacerbating cardiac dysfunction and remodeling^10,17^. Glucose-to-lymphocyte ratio (GLR), as a novel inflammatory marker, is associated with the mortality risk of various inflammatory diseases, including acute myocardial infarction (AMI), chronic obstructive pulmonary disease (COPD), and acute respiratory distress syndrome (ARDS), and it shows remarkable prognostic value^18–20^. However, there are currently no studies in the available literature examining the association between GLR and mortality risk in patients with heart failure.

Therefore, to investigate the relationship between GLR and mortality risk in patients with heart failure and the predictive value of GLR for mortality in this population, we conducted a retrospective cohort study. Our aim was to find a useful marker that can predict the prognosis of patients with heart failure.

## Methods

### Data source

Data for this retrospective cohort study were sourced from the publicly available Medical Information Mart for Intensive Care IV (MIMIC-IV, version 3.0) database, which includes information on over 50,000 intensive care unit (ICU) admissions at Beth Israel Deaconess Medical Center in Boston from 2008 to 2022^21,22^. The author Gang Wu was granted access to the database after completing the necessary online training and certification procedure (certification number: 62773844). The use of the MIMIC-IV database was authorized by the institutional review boards at both the Massachusetts Institute of Technology and Beth Israel Deaconess Medical Center. Since the data is available to the public, ethical clearance for the study was not necessary, and since the research was retrospective, informed consent was not needed.

### Study design and population

**Fig. 1:**
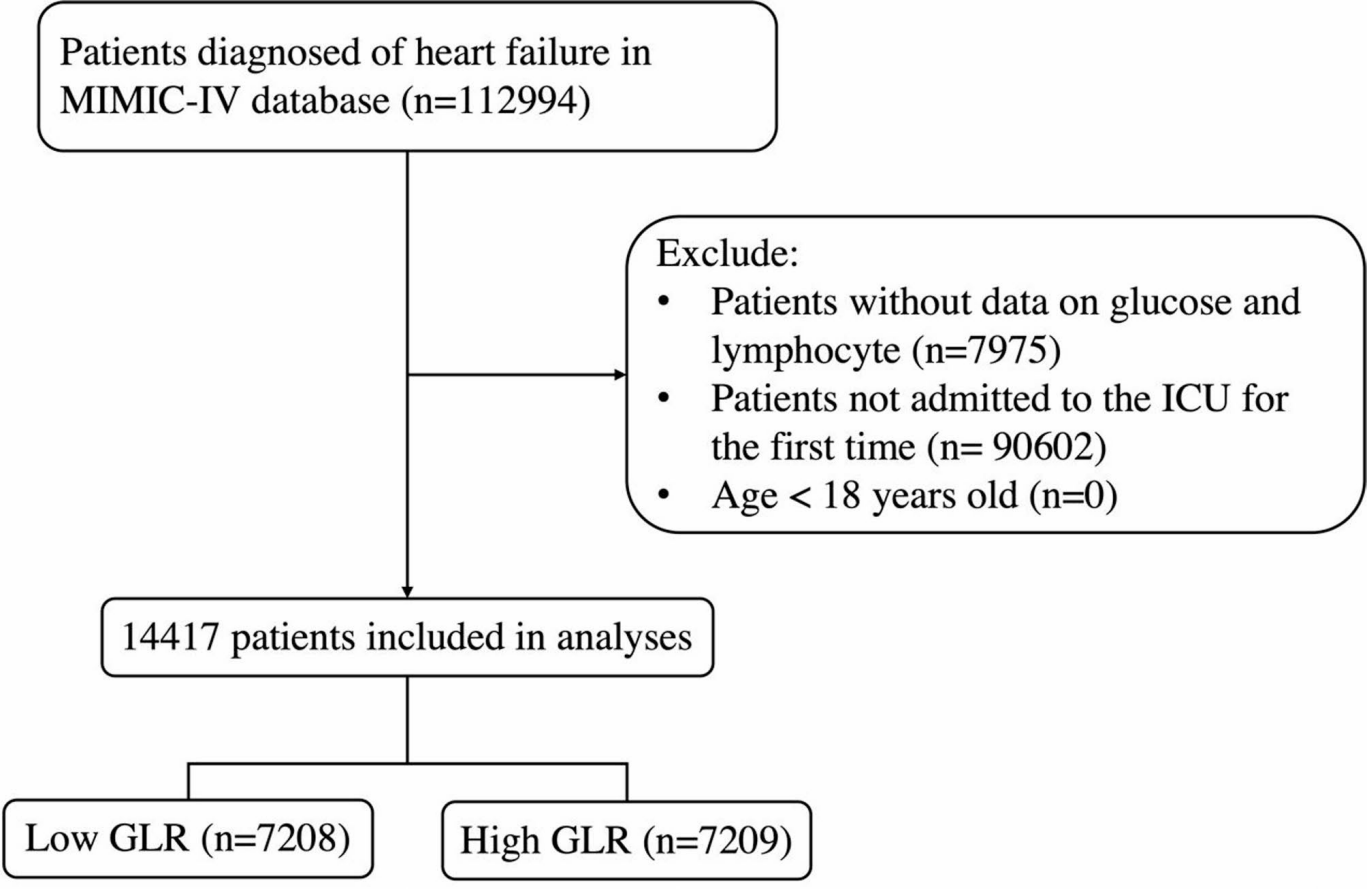
Flowchart of the study. *GLR* glucose-to-lymphocyte ratio.

Figure 1 presents the study’s workflow. We assembled a cohort of 112,994 individuals diagnosed with heart failure, identified through the search terms “heart failure” using the International Classification of Diseases, Ninth and Tenth Revisions (ICD-9 and ICD-10) coding systems. The exclusion criteria were as follows: (1) individuals missing information on glucose and lymphocyte; (2) patients who had multiple ICU admissions due to heart failure, with only data from the initial admission being analyzed; and (3) age < 18 years old. Ultimately, a total of 14,417 participants were retained in the final cohort and categorized into two groups based on the median value of the GLR (median GLR value = 143.35, Low GLR ≤ 143.35, High GLR > 143.35).

### Data extraction

We extracted study variables using Structured Query Language (SQL) via Navicat Premium (Version 16.2.9). These variables included: age, sex, weight, heart rate (HR), systolic blood pressure (SBP), diastolic blood pressure (DBP), mean arterial pressure (MAP), respiratory rate (RR), oxygen saturation (SpO2), Sequential Organ Failure Assessment (SOFA) score, Charlson Comorbidity Index (CCI), red blood cell (RBC) count, red cell distribution width (RDW), hematocrit, hemoglobin, white blood cell (WBC) count, neutrophils, lymphocytes, monocytes, platelets, creatinine, bicarbonate, blood urea nitrogen (BUN), sodium, potassium, calcium, anion gap, glucose, myocardial infarction, atrial fibrillation, hypertension, cerebrovascular diseases, diabetes mellitus, renal diseases, and the use of various medications, including beta blockers, angiotensin-converting enzyme inhibitors (ACEI), angiotensin II receptor blockers (ARB), angiotensin receptor-neprilysin inhibitors (ARNI), calcium channel blockers (CCB), diuretics, antidiabetics, and epinephrine. Additional variables included the length of hospital stay (LOS), which encompassed both hospital LOS and intensive care unit (ICU) LOS, as well as 30-day mortality rate and 365-day mortality rate. To minimize potential confounding effects, all variables analyzed in our study were derived from blood samples collected within the first 24 h of ICU admission for heart failure patients.

### Calculation of GLR

The formula that was used to calculate the GLR was as follows: glucose (mg/dL) divided by lymphocyte count (K/uL)^18^.

### Outcomes

The clinical outcome was 30-day mortality rates and 365-day mortality rates. The mortality data were documented in the MIMIC-IV database. We calculated the survival time for each patient by subtracting the discharge date from the date of death.

### Statistical analysis

Measurement data that followed a normal distribution were characterized using mean ± standard deviation (Mean ± SD), while a t-test was employed to compare two groups. For data with a skewed distribution, results were presented as median and quartiles [M (Q1, Q3)], with the Wilcoxon rank sum test applied for comparisons. Categorical data distributions were described using frequency with composition ratios [N (%)], and comparisons were made using the chi-square test or Fisher’s exact test.

To investigate the relationship between GLR and outcome incidence rates in heart failure patients, both univariate and multivariate COX proportional hazards models were constructed. The selection of confounding variables was primarily based on clinical relevance and univariate analysis results (*p* < 0.1). Specifically, our adjustment model included the following covariates: gender, age, weight, SOFA score, WBC, RBC, RDW, hemoglobin, platelet, creatinine, BUN, anion gap, sodium, potassium, calcium, HR, SBP, DBP, RR, SpO2, myocardial infarction, cerebrovascular disease, diabetes, hypertension, atrial fibrillation, beta blocker use, ACEI/ARB/ARNI use, antidiabetics use, and diuretic use. We assessed multicollinearity among variables by calculating the variance inflation factor (VIF) for all covariates in the final model. A VIF < 5 indicated that multicollinearity was unlikely to substantially influence the key conclusions of our study. Restricted cubic spline (RCS) curves were used to illustrate the relationship between GLR levels and the risk of 30-day and 365-day mortality. To account for potential confounding factors, we adjusted for the following covariates: sex, age, weight, SOFA score, WBC, RBC, RDW, hemoglobin, platelet count, creatinine, BUN, anion gap, sodium, potassium, HR, SBP, DBP, RR, SpO₂, as well as comorbidities (myocardial infarction, cerebrovascular disease, diabetes, hypertension, atrial fibrillation) and medications (beta-blockers, ACEI/ARB/ARNI, antidiabetic drugs, diuretics). The selection of confounding variables was based on clinical relevance and established scientific literature. Additionally, the Kaplan-Meier (K-M) survival curve assessed the correlation between GLR levels and the survival probability of patients with heart failure. GLR, glucose levels, and lymphocyte counts were evaluated for their forecasting potential using receiver operating characteristic (ROC) curve analysis. The DeLong test was used to compare the area under the curve (AUC) of different models. The primary metrics for evaluation included hazard ratios (HRs) and confidence intervals (CIs), with *P* < 0.05 indicating statistical significance.

Statistics analyses were completed via SPSS (version 26.0, IBM Corporation, USA) and R software (version 4.4.1).

## Results

### Characteristics of heart failure patients

Table 1 presents the characteristics of heart failure patients stratified by different GLR levels. Among them, 2,918 patients (20.24%) died within 30 days, while 5,912 patients (41.01%) succumbed within 365 days. The average age across the cohort was 72.77 years, with males comprising 8,037 (55.75%) of the population. Comparative analysis revealed that the median values for glucose, lymphocytes, and GLR differed significantly between the low and high GLR level groups. Specifically, the median glucose levels were 132 mg/dL in the low GLR group versus 189 mg/dL in the high GLR group; median lymphocyte counts were 1.67 K/uL versus 0.73 K/uL; and median GLR values were 85.03 versus 256.28, respectively.

**Table 1:** Characteristics of heart failure patients with varying levels of GLR.

Variables	Total (*n* = 14417)	Low GLR (*n* = 7208)	High GLR (*n* = 7209)	*P*
Age, years, Mean ± SD	72.77 ± 13.56	71.98 ± 14.03	73.55 ± 13.02	< 0.001
Gender, n (%)				0.280
Male	8037 (55.75)	3986 (49.60)	4051 (50.40)	
Female	6380 (44.25)	3222 (50.50)	3158 (49.50)	
Weight, kg, M (Q_1_, Q_3_)	80.00 (66.40, 95.45)	80.38 (67.00, 96.08)	79.50 (66.00, 94.87)	0.011
HR, bpm, M (Q_1_, Q_3_)	82.74 (73.08, 95.04)	81.69 (72.66, 93.34)	84.00 (73.40, 96.69)	< 0.001
SBP, mmHg, M (Q_1_, Q_3_)	112.86 (103.93, 124.90)	112.38 (103.89, 123.30)	113.44 (103.96, 126.22)	< 0.001
DBP, mmHg, M (Q_1_, Q_3_)	60.52 (54.08, 68.20)	60.25 (53.96, 68.00)	60.82 (54.19, 68.38)	0.085
MBP, mmHg, M (Q_1_, Q_3_)	75.21 (69.29, 82.46)	75.09 (69.26, 82.08)	75.33 (69.30, 82.87)	0.262
RR, bpm, M (Q_1_, Q_3_)	19.61 (17.35, 22.38)	19.26 (17.14, 21.88)	19.96 (17.62, 22.84)	< 0.001
Spo2, %, Mean ± SD	96.49 ± 2.50	96.65 ± 2.39	96.32 ± 2.60	< 0.001
SOFA scores, M (Q_1_, Q_3_)	5.00 (3.00, 7.00)	5.00 (3.00, 7.00)	5.00 (3.00, 8.00)	< 0.001
RBC, K/µL, M (Q_1_, Q_3_)	3.50 (3.01, 3.90)	3.50 (3.07, 3.93)	3.45 (2.95, 3.86)	< 0.001
RDW, %, M (Q_1_, Q_3_)	15.70 (14.30, 16.90)	15.50 (14.10, 16.60)	15.80 (14.40, 17.20)	< 0.001
Hematocrit, %, M (Q_1_, Q_3_)	33.90 (29.70, 38.80)	34.30 (30.30, 39.00)	33.60 (29.10, 38.50)	< 0.001
Hemoglobin, g/dL, M (Q_1_, Q_3_)	10.90 (9.40, 12.50)	11.00 (9.70, 12.60)	10.70 (9.20, 12.40)	< 0.001
WBC, K/µL, M (Q_1_, Q_3_)	12.70 (9.00, 17.70)	13.30 (9.50, 18.50)	12.00 (8.50, 16.90)	< 0.001
Neutrophils, K/µL, M (Q_1_, Q_3_)	8.99 (6.02, 13.30)	8.84 (5.96, 13.08)	9.13 (6.11, 13.54)	0.014
Monocytes, K/µL, M (Q_1_, Q_3_)	0.60 (0.37, 0.92)	0.65 (0.42, 0.97)	0.55 (0.33, 0.87)	< 0.001
Platelet, K/µL, M (Q_1_, Q_3_)	214.00 (158.00, 283.00)	217.00 (162.00, 287.00)	209.00 (154.00, 279.00)	< 0.001
Creatinine, mg/dL, M (Q_1_, Q_3_)	1.50 (1.00, 2.40)	1.30 (0.90, 2.10)	1.70 (1.10, 2.80)	< 0.001
Bicarbonate, mmol/L, M (Q_1_, Q_3_)	25.00 (22.00, 28.00)	25.00 (22.00, 28.00)	25.00 (22.00, 28.00)	0.040
BUN, mmol/L, M (Q_1_, Q_3_)	32.00 (21.00, 51.00)	27.00 (18.00, 44.00)	37.00 (24.00, 58.00)	< 0.001
Sodium, mmol/L, M (Q_1_, Q_3_)	140.00 (137.00, 142.00)	140.00 (137.00, 142.00)	140.00 (136.00, 143.00)	< 0.001
Potassium, mmol/L, M (Q_1_, Q_3_)	4.60 (4.20, 5.30)	4.60 (4.20, 5.10)	4.70 (4.20, 5.40)	< 0.001
Calcium, mmol/L, M (Q_1_, Q_3_)	8.70 (8.30, 9.20)	8.70 (8.30, 9.20)	8.70 (8.20, 9.20)	0.024
Anion gap, mmol/L, M (Q_1_, Q_3_)	16.00 (14.00, 20.00)	16.00 (13.00, 19.00)	17.00 (15.00, 21.00)	< 0.001
Myocardial infarction, n (%)				0.005
NO	9824 (68.14)	4990 (69.23)	4834 (67.06)	
Yes	4593 (31.86)	2218 (30.77)	2375 (32.94)	
Hypertension, n (%)				< 0.001
NO	11,278 (78.23)	5497 (76.26)	5781 (80.19)	
Yes	3139 (21.77)	1711 (23.74)	1428 (19.81)	
Atrial fibrillation, n (%)				0.006
NO	7014 (48.65)	3590 (49.81)	3424 (47.50)	
Yes	7403 (51.35)	3618 (50.19)	3785 (52.50)	
Cerebrovascular disease, n (%)				0.007
NO	12,519 (86.83)	6204 (86.07)	6315 (87.60)	
Yes	1898 (13.17)	1004 (13.93)	894 (12.40)	
Diabetes, n (%)				< 0.001
No	8021 (55.64)	4607 (63.92)	3414 (47.36)	
Yes	6396 (44.36)	2601 (36.08)	3795 (52.64)	
Renal disease, n (%)				< 0.001
NO	8102 (56.20)	4438 (61.57)	3664 (50.83)	
Yes	6315 (43.80)	2770 (38.43)	3545 (49.17)	
Beta blocker, n (%)				< 0.001
NO	3292 (22.83)	1471 (20.41)	1821 (25.26)	
Yes	11,125 (77.17)	5737 (79.59)	5388 (74.74)	
ACEI/ARB/ARNI, n (%)				< 0.001
NO	8979 (62.28)	4246 (58.91)	4733 (65.65)	
Yes	5438 (37.72)	2962 (41.09)	2476 (34.35)	
CCB, n (%)				0.039
NO	10,178 (70.60)	5145 (71.38)	5033 (69.82)	
Yes	4239 (29.40)	2063 (28.62)	2176 (30.18)	
Diuretics, n (%)				0.857
NO	2981 (20.68)	1486 (20.62)	1495 (20.74)	
Yes	11,436 (79.32)	5722 (79.38)	5714 (79.26)	
Antidiabetics, n (%)				< 0.001
NO	3283 (22.77)	1851 (25.68)	1432 (19.86)	
Yes	11,134 (77.23)	5357 (74.32)	5777 (80.14)	
Epinephrine, n (%)				< 0.001
NO	8908 (61.79)	4573 (63.44)	4335 (60.13)	
Yes	5509 (38.21)	2635 (36.56)	2874 (39.87)	
LOS hospital, day, M (Q_1_, Q_3_)	8.12 (4.92, 13.92)	7.88 (4.85, 13.37)	8.60 (5.00, 14.62)	< 0.001
LOS ICU, day, M (Q_1_, Q_3_)	2.34 (1.27, 4.67)	2.23 (1.24, 4.22)	2.54 (1.33, 5.09)	< 0.001
30-day mortality, n (%)				< 0.001
NO	11,499 (79.76)	6139 (85.17)	5359 (74.33)	
Yes	2918 (20.24)	1069 (14.83)	1850 (25.67)	
365-day mortality, n (%)				< 0.001
NO	8505 (58.99)	4829 (67.00)	3675 (50.98)	
Yes	5912 (41.01)	2379 (33.00)	3534 (49.02)	

*SD* standard deviation, *M* median, *Q*_*1*_ 1 st quartile, *Q*_*3*_ 3 st quartile, *HR* heart rate, *SBP* systolic blood pressure, *DBP* diastolic blood pressure, *MBP* mean blood pressure, *RR* respiratory rate, *SOFA* sequential organ failure assessment, *CCI* charlson comorbidity index, *RBC* red blood cell, *RDW* red cell distribution width, *WBC* white blood cell, *BUN* blood urea nitrogen, *LOS* length of stay, *ICU* intensive care unit, *ACEI* angiotensin-converting enzyme inhibitor, *ARB* angiotensin II receptor blockers, *ARNI* angiotensin receptor-neprilysin inhibitor, *CCB* calcium channel blockers.

### The detection of nonlinear relationships

**Fig. 2:**
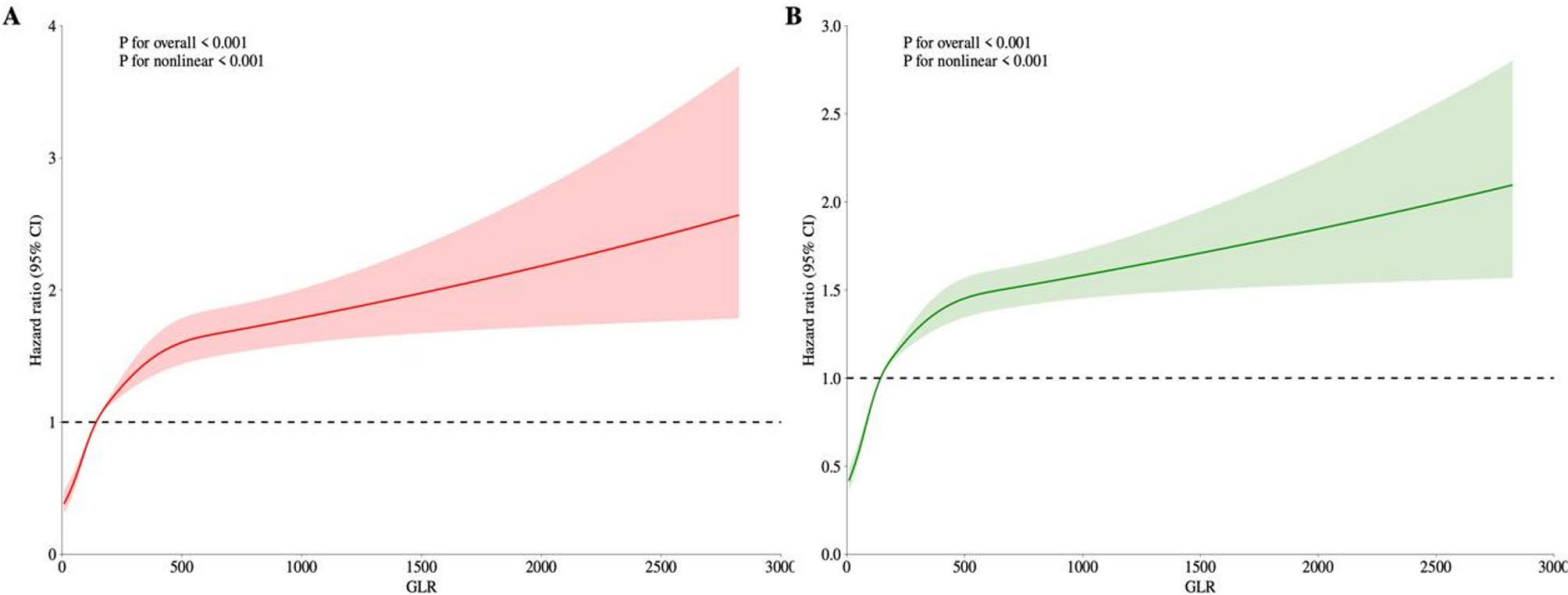
Relationship between GLR levels and mortality at 30 days (**A**) and 365 days (**B**) in heart failure patients by RCS analysis. *GLR* glucose-to-lymphocyte ratio.

Figure 2 presents the RCS curve illustrating the association between GLR levels and the risk of 30-day and 365-day mortality. The data shows that as GLR levels rise, the hazard ratio also increases. Furthermore, nonlinear testing confirmed that the RCS curve is nonlinear (*P* < 0.001).

### Association between GLR level with clinic outcomes in heart failure patients

Univariate and multivariate Cox regression analyses revealed a significant independent association between higher GLR levels and increased 30-day and 365-day mortality in heart failure patients (*P <* 0.001) (Table 2). This association persisted even after adjusting for demographic characteristics, vital signs, comorbidity, medication use, and comprehensive laboratory parameters (*P* < 0.05). We assessed multicollinearity among variables by calculating the VIF for all covariates in the final model. The results demonstrate that all VIF values < 5, suggesting that multicollinearity is unlikely to substantially influence the key conclusions of our study (i.e., the association between GLR levels and outcomes in heart failure patients). (Supplementary Table S1).

**Table 2:** Relationship between GLR levels and mortality at 30 days and 365 days in a Cox regression model.

GLR	Model1		Model2		Model3		Model4	
	HR (95%CI)	*P*	HR (95%CI)	*P*	HR (95%CI)	*P*	HR (95%CI)	*P*
30-day mortality								
Continuous variable	1.01 (1.01, 1.01)	< 0.001	1.01 (1.01, 1.01)	< 0.001	1.01 (1.01, 1.01)	0.005	1.01 (1.01, 1.01)	0.031
Categorical variable								
≤ 143.35	1.00 (Reference)		1.00 (Reference)		1.00 (Reference)		1.00 (Reference)	
> 143.35	1.78 (1.66, 1.92)	< 0.001	1.58 (1.47, 1.71)	< 0.001	1.47 (1.36, 1.59)	< 0.001	1.37 (1.26, 1.48)	< 0.001
365-day mortality								
Continuous variable	1.01 (1.01, 1.01)	< 0.001	1.01 (1.01, 1.01)	< 0.001	1.01 (1.01, 1.01)	< 0.001	1.01 (1.01, 1.01)	< 0.001
Categorical variable								
≤ 143.35	1.00 (Reference)		1.00 (Reference)		1.00 (Reference)		1.00 (Reference)	
> 143.35	1.70 (1.61,1.79)	< 0.001	1.57 (1.49, 1.65)	< 0.001	1.43 (1.35, 1.51)	< 0.001	1.34 (1.27, 1.42)	< 0.001

*HR* Hazard Ratio, *CI* Confidence Interval.

Model 1: Crude.

Model 2: Adjust: gender, age, weight, SOFA.

Model 3: Adjust: gender, age, weight, SOFA, WBC, RBC, RDW, hemoglobin, platelet, creatinine, BUN, anion gap, sodium, potassium, calcium.

Model 4: Adjust: gender, age, weight, SOFA, WBC, RBC, RDW, hemoglobin, platelet, creatinine, BUN, anion gap, sodium, potassium, calcium, HR, SBP, DBP, RR, SpO2, myocardial infarction, cerebrovascular disease, diabetes, hypertension, atrial fibrillation, beta blocker, ACEI/ARB/ARNI, antidiabetics, diuretic.

### Kaplan-Meier survival analysis

**Fig. 3:**
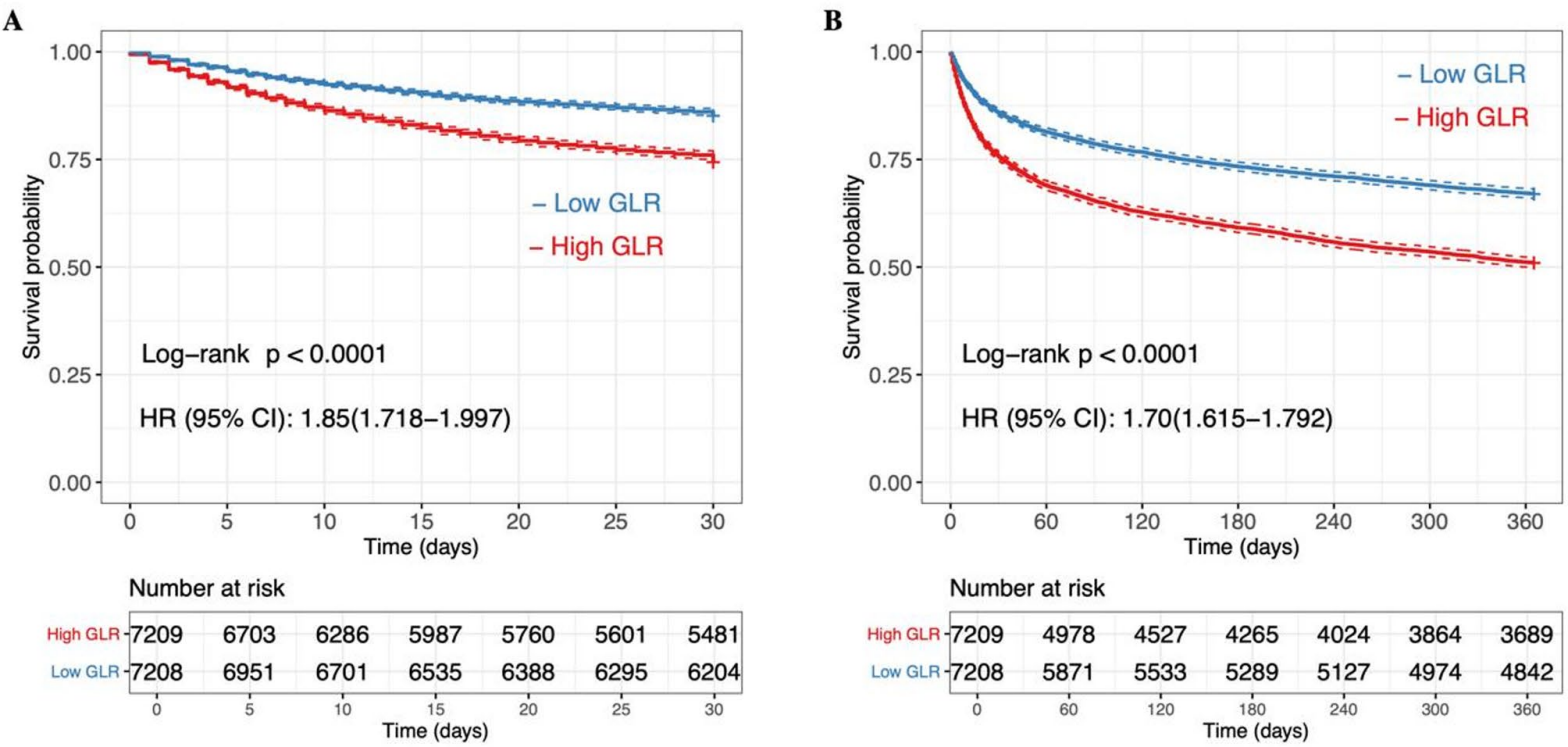
The K-M curve of association between GLR levels and mortality at 30 days (**A**) and 365 days (**B**) in heart failure patients. *GLR* glucose-to-lymphocyte ratio.

We also plotted the K-M survival curve to illustrate the relationship between GLR levels and mortality at 30 days and 365 days in heart failure patients (Fig. 3). The K-M survival curve revealed that the 30-day and 365-day mortality rates for heart failure patients with low GLR levels were significantly lower compared to those with high GLR levels (*P* < 0.0001). This indicates a statistically significant difference in survival probability between the two groups.

### Stratified analyses

**Fig. 4:**
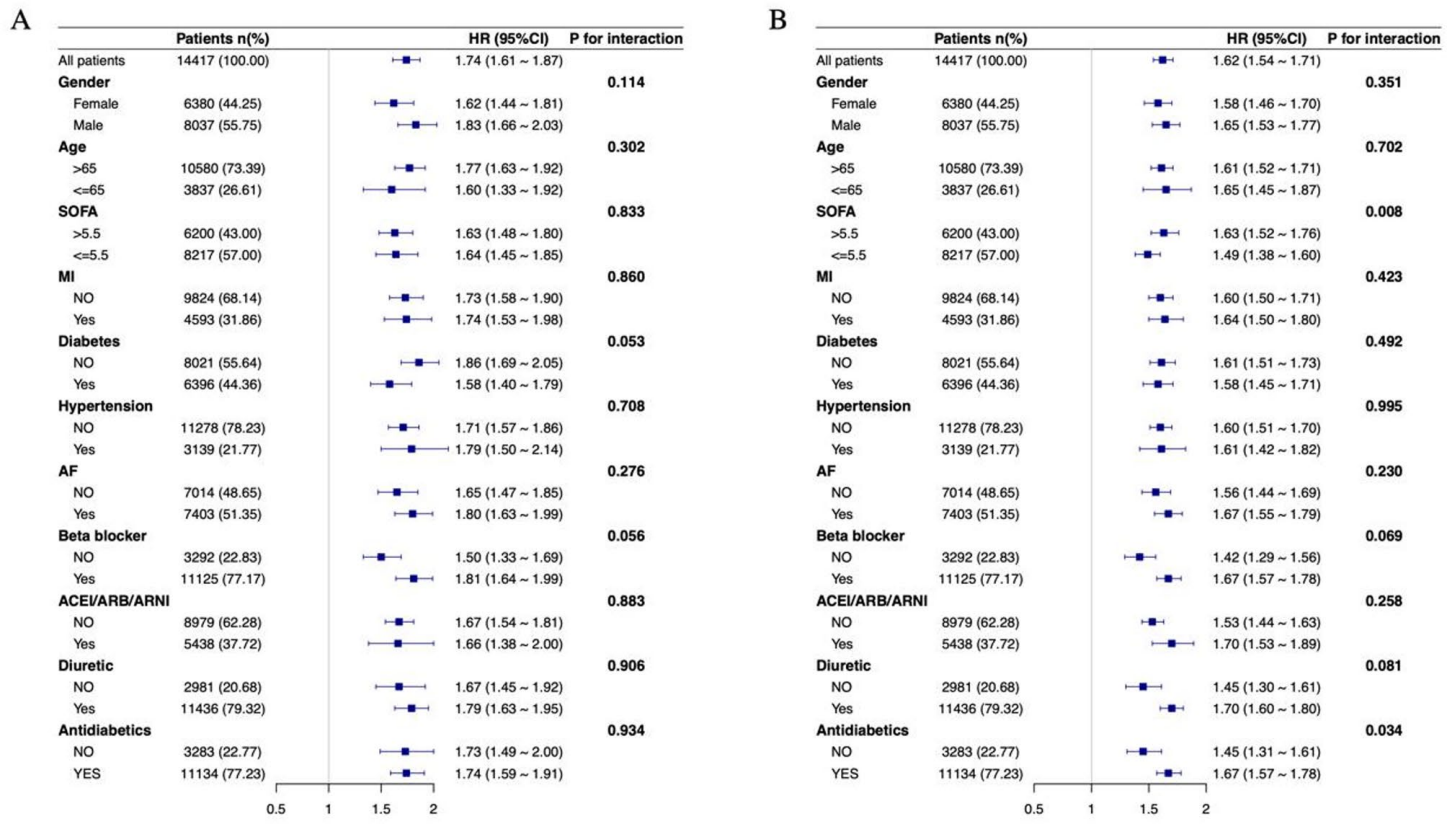
Stratified analyses of the relationship between GLR and mortality at 30 days (**A**) and 365 days (**B**) in heart failure patients. *SOFA* sequential organ failure assessment, *MI* myocardial infarction, *AF* atrial fibrillation.

To further elucidate whether the relationship between GLR and clinical outcomes in heart failure patients is influenced by different conditions, we conducted a subgroup analysis. The data were analyzed based on factors such as gender, age, SOFA score, myocardial infarction, diabetes, hypertension, atrial fibrillation, β-blockers, ACEI/ARB/ARNI, diuretics, and antidiabetics. Figure 4A shows that for the 30-day outcome, no significant interactions were found between GLR and the other subgroups (interaction p-values: 0.053–0.934). However, for the 365-day outcome, significant interactions were noted for SOFA (*P* = 0.008), and antidiabetics (*P* < 0.034), with no substantial interactions between GLR and the remaining subgroups (interaction p-values: 0.069–0.995) (Fig. 4B). This suggests that the association between GLR and clinical outcomes in heart failure patients was largely consistent across most of the examined subgroups in this study, except for SOFA score and antidiabetic medication use at 365 days.

### Predictive value of GLR on clinic outcomes in heart failure patients

**Fig. 5:**
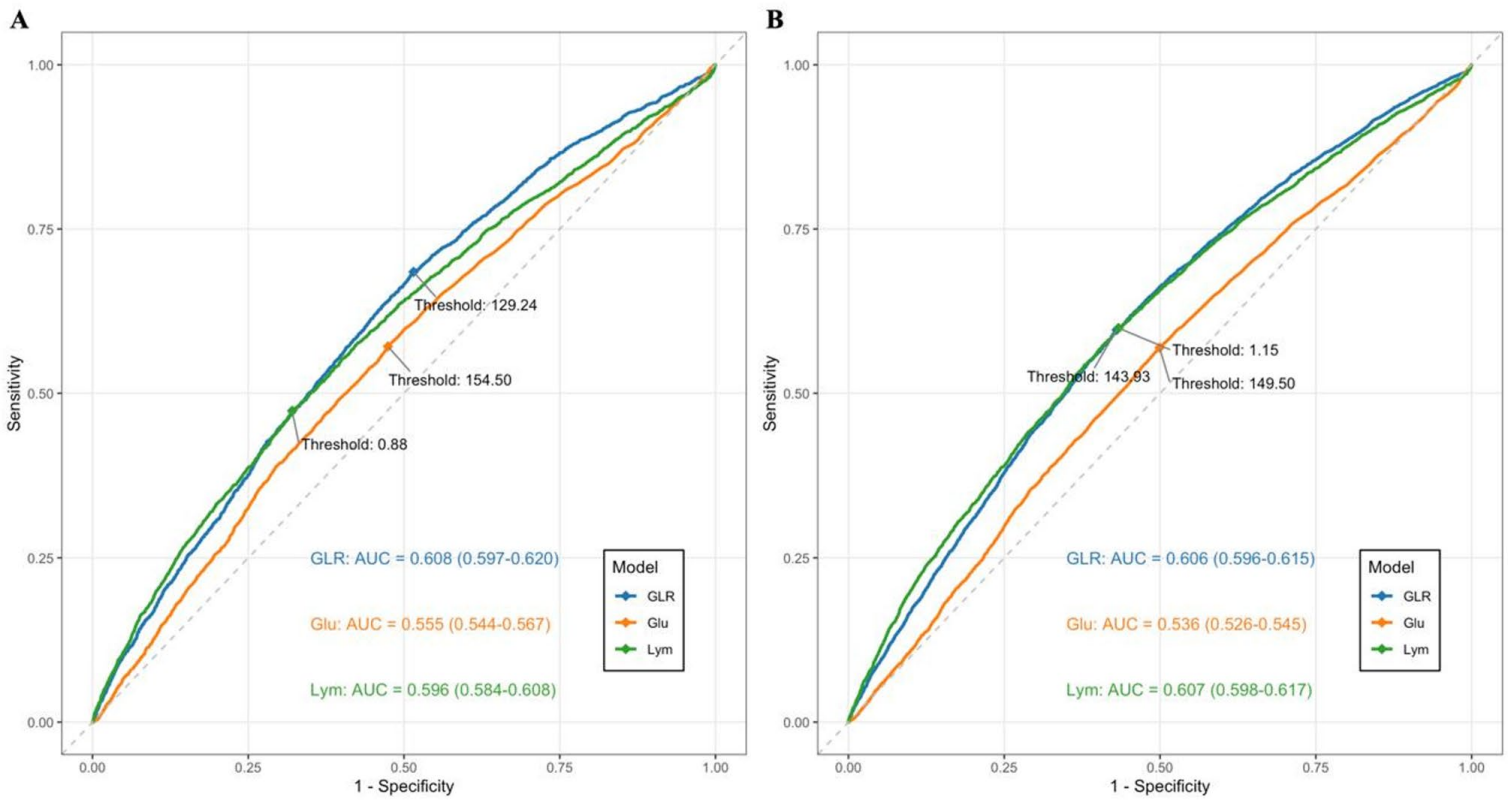
ROC curve analysis of surrogate markers for predict 30-day (**A**) and 365-day (**B**) mortality in heart failure patients. *AUC* area under the curve, *GLR* glucose-to-lymphocyte ratio, *Glu* glucose, *Lym* lymphocyte.

Figure 5 illustrates the ROC curves for predicting 30-day and 365-day mortality in patients with heart failure admitted to the ICU, using GLR, glucose, and lymphocytes. For the prediction of 30-day mortality in heart failure patients, the GLR curve consistently outperformed both the glucose and lymphocyte curves. In terms of predicting 365-day mortality, the GLR curve remained superior to the glucose curve, while being comparable to the lymphocyte curve (Table 3). Overall, these findings suggest that GLR may possess a greater predictive value than either glucose or lymphocytes.

**Table 3:** Area under the ROC curve comparison of glucose, lymphocytes, and GLR.

Model1	Model2	AUC1	AUC2	Diff	SE	Z	*P* ^*^
30-day							
GLR	Glucose	0.608	0.555	0.053	0.006	8.439	< 0.001
GLR	Lymphocyte	0.608	0.596	0.012	0.003	3.543	< 0.001
Glu	Lymphocyte	0.555	0.596	−0.040	0.009	−4.586	< 0.001
365-day							
GLR	Glucose	0.606	0.536	0.070	0.005	14.482	< 0.001
GLR	Lymphocyte	0.606	0.607	−0.002	0.003	−0.589	0.556
Glu	Lymphocyte	0.536	0.607	−0.071	0.007	−10.428	< 0.001

*DeLong’s test. AUC: area under the curve. *Diff* difference, *SE* standard error, *GLR* glucose-to-lymphocyte ratio.

## Discussion

To the best of our knowledge, this study is the first retrospective investigation of the relationship between GLR levels and mortality risk in critically ill patients with heart failure. According to our findings, these people have a higher chance of dying within 30 days and within 365 days if their GLR levels are higher. It is noteworthy that this relationship remains statistically significant even after taking into account potential confounding factors. Furthermore, this work provides a clear and simple methodology to improve mortality risk stratification in critically ill patients with heart failure. Importantly, our research suggests that the predictive ability of GLR for mortality in patients with heart failure may exceed that of glucose or lymphocyte levels. Meanwhile, GLR levels provide predictive value for both 30-day and 365-day mortality in patients with heart failure, with AUC values of 0.608 and 0.606, respectively.

GLR, a distinctive inflammatory biomarker that integrates blood glucose levels and lymphocyte counts, demonstrated remarkable predictive capacity for both short-term and long-term mortality in certain diseases. Previous studies have reported that GLR is a prognostic marker for a variety of tumors^23–25^. Furthermore, Tianyang et al. demonstrated the higher predictive value of GLR for in-hospital mortality in acute exacerbation of COPD compared to neutrophil-lymphocyte ratio, platelet-lymphocyte ratio, and systemic immune-inflammation index^19^. Also, another study indicated that GLR serves as an independent prognostic marker for critically ill patients suffering from ARDS^20^. A recent retrospective cohort study revealed that the predictive ability of GLR for in-hospital mortality among patients with AMI may surpass that of glucose or lymphocyte levels^18^. In a similar vein, our current investigation revealed that elevated levels of GLR correlate with a higher risk of mortality in individuals with heart failure. We also evaluated the predictive capacity of GLR and compared it to that of glucose and lymphocyte counts. The findings suggested that GLR’s predictive efficacy may surpass the other two markers. Notably, we observed a nonlinear relationship between GLR levels and mortality risk at both 30-day and 365-day follow-ups, suggesting a threshold effect where mortality risk escalates sharply beyond a critical GLR value. Thus, our results may provide valuable insights supporting that GLR is a promising predictor for heart failure outcomes.

The mechanism by which elevated GLR increases the risk of mortality in heart failure patients is likely multifactorial, reflecting the complex interplay between dysregulated immunity and metabolic dysfunction inherent in the heart failure syndrome. Our findings suggest that both components of the GLR ratio—lymphopenia and hyperglycemia—are significant contributors to poor outcomes, and their combination may provide a more integrated reflection of the underlying pathological state than either marker alone. The potential pathophysiological links between elevated GLR and heightened mortality in patients with heart failure may be explained as follows: On the one hand, heart failure typically results in a systemic inflammatory response, with a decreased lymphocyte count frequently observed during this response^26,27^. This is consistent with our findings. The factors contributing to the reduction in lymphocyte count during systemic inflammation can be attributed to increased lymphocyte apoptosis^28^; diminished lymphocyte proliferation and differentiation; and the redistribution of lymphocytes within lymphopoietic systems^29^. On the other hand, in the early stages of heart failure, glucose utilization increases, while free fatty acid utilization can be unchanged or only mildly increased^8^. However, since insulin resistance can be present in severe heart failure, glucose utilization decreases, raising blood glucose^30–34^. Just as we found out. Mechanisms contributing to cardiac insulin resistance in heart failure may involve diminished mitochondrial oxidative capability^35^, increased rates of fatty acid metabolism, and decreased glucose metabolism^8^. These alterations may influence the quantity of lymphocytes and concentration of glucose via various pathways, thereby impacting GLR.

However, lymphopenia and hyperglycemia in heart failure are not isolated phenomena but are often interconnected and mutually reinforcing^36^. Chronic inflammation drives both lymphocyte depletion/apoptosis and insulin resistance. Conversely, hyperglycemia can directly impair lymphocyte function, proliferation, and survival through mechanisms involving oxidative stress, glycation of surface receptors, and altered metabolism within immune cells^37,38^. The neurohormonal activation characteristic of heart failure (sympathetic overdrive, RAAS, cortisol) simultaneously promotes hyperglycemia and suppresses lymphocyte numbers and function^39,40^. Therefore, an elevated GLR likely signifies a more profound and dysregulated state of systemic inflammation, metabolic stress, and neurohormonal activation – a “perfect storm” contributing to accelerated myocardial damage, increased vulnerability to decompensation triggers (like infection), and ultimately, higher mortality risk^41,42^. This synergy explains why GLR’s predictive power surpasses its individual components, and why we observed a nonlinear mortality curve: once GLR exceeds a threshold, the combined metabolic-immune derangement overwhelms compensatory mechanisms.

In summary, While our findings highlight the potential utility of GLR as a readily available prognostic biomarker in heart failure, further investigations are required to prospectively validate its predictive value, elucidate the causal relationships and detailed molecular mechanisms linking GLR components to specific cardiac pathologies, and explore whether targeting these pathways (e.g., modulating inflammation, improving glycemic control in specific subsets) can improve outcomes in high-GLR heart failure patients.

The findings provide strong evidence regarding how GLR correlates with mortality risk among individuals with heart failure. We observed a nonlinear relationship between GLR levels and the risk of mortality at both 30-day and 365-day, offering support for its clinical relevance. Firstly, GLR is a simple, cost-effective, and widely accessible biochemical marker that can assist in assessing prognosis and stratifying risk in heart failure patients. This facilitates the creation of personalized and precise medical decisions. Secondly, if a patient presents with abnormal GLR values, it is crucial for clinicians to investigate the underlying pathophysiological factors, such as inflammation and immune responses, and implement timely interventions to enhance patient outcomes. Furthermore, upcoming clinical trials might explore GLR as a potential alternative indicator for evaluating treatment efficacy and investigating innovative strategies to strengthen heart failure outcomes through the modulation of GLR levels. This could open new avenues for both pharmaceutical and non-pharmaceutical interventions. In conclusion, our findings lay a solid evidence-based framework for incorporating GLR into the secondary prevention strategies for heart failure.

This study has to acknowledge several limitations in its findings. The retrospective design may harbor residual confounders despite the application of regression models and sensitivity analyses. While GLR was evaluated at the time of admission utilizing baseline blood counts and initial biochemical indicators to reduce the influence of treatment, the effects of extended pharmacological therapies on heart failure outcomes require further clarification. The absence of variables such as inflammation, NT-proBNP, and cardiac size restricts a comprehensive analysis of mechanisms linking these factors to GLR. Selection bias is also a concern due to reliance on the MIMIC-IV database, potentially skewing the representation of the outpatient heart failure population and neglecting confounding lifestyle and genetic factors. Furthermore, the inability to continuously measure GLR limits the assessment of its change patterns and prognostic relevance. Given that the findings are based on a single-center study, their external validity is constrained. Future investigations involving multi-center, multi-ethnic cohorts are essential for validating and broadening these results, enhancing our understanding of GLR’s role in heart failure outcomes.

## Conclusion

Our study found a non-linear relationship between GLR levels and mortality at both 30-day and 365-day among patients with heart failure, indicating that higher GLR levels are linked to an increased risk of death. These findings suggest that GLR could be an important predictor for heart failure prognosis, potentially assisting in the identification and management of high-risk populations in clinical practice. However, additional research is necessary to clarify the causal relationship between GLR levels and mortality risk in heart failure patients.

## Electronic supplementary material

Below is the link to the electronic supplementary material.

Supplementary Material 1

Supplementary Material 2

## Data Availability

The datasets used in this study were publicly available, derived from the MIMIC-IV database (version 3.0), and are available at https://mimic.mit.edu/.

## References

[1] Dokainish, H. et al. Heart failure in africa, asia, the middle East and South america: the INTER-CHF study. *Int. J. Cardiol.* **204**, 133–141 (2016). 26657608 10.1016/j.ijcard.2015.11.183

[2] Dokainish, H. et al. Global mortality variations in patients with heart failure: results from the international congestive heart failure (INTER-CHF) prospective cohort study. *Lancet Glob Health*. **5** (7), e665–e672 (2017). 28476564 10.1016/S2214-109X(17)30196-1

[3] Cook, C., Cole, G., Asaria, P., Jabbour, R. & Francis, D. P. The annual global economic burden of heart failure. *Int. J. Cardiol.* **171** (3), 368–376 (2014). 24398230 10.1016/j.ijcard.2013.12.028

[4] Michels da Silva, D., Langer, H. & Graf, T. Inflammatory and molecular pathways in heart failure-ischemia, HFpEF and transthyretin cardiac amyloidosis. *Int. J. Mol. Sci.* **20**, 9 (2019). 10.3390/ijms20092322 PMC6540104 31083399

[5] Xiaojing, C., Yanfang, L., Yanqing, G. & Fangfang, C. Thymopentin improves cardiac function in older patients with chronic heart failure. *Anatol. J. Cardiol.* **17** (1), 24–30 (2017). 27564775 10.14744/AnatolJCardiol.2016.6692 PMC5324858

[6] Liu, L. et al. Association between haemoglobin, albumin, lymphocytes, and platelets and mortality in patients with heart failure. *ESC Heart Fail.* **11** (2), 1051–1060 (2024). 38243382 10.1002/ehf2.14662 PMC10966267

[7] Kong, P., Christia, P. & Frangogiannis, N. G. The pathogenesis of cardiac fibrosis. *Cell. Mol. Life Sci.* **71** (4), 549–574 (2014). 23649149 10.1007/s00018-013-1349-6 PMC3769482

[8] Lopaschuk, G. D., Karwi, Q. G., Tian, R., Wende, A. R. & Abel, E. D. Cardiac energy metabolism in heart failure. *Circ. Res.* **128** (10), 1487–1513 (2021). 33983836 10.1161/CIRCRESAHA.121.318241 PMC8136750

[9] Yang, Z., Gong, H., Kan, F. & Ji, N. Association between the triglyceride glucose (TyG) index and the risk of acute kidney injury in critically ill patients with heart failure: analysis of the MIMIC-IV database. *Cardiovasc. Diabetol.* **22** (1), 232 (2023). 37653418 10.1186/s12933-023-01971-9 PMC10472684

[10] Nakamura, K. et al. Pathophysiology and treatment of diabetic cardiomyopathy and heart failure in patients with diabetes mellitus. *Int. J. Mol. Sci.* **23**, 7 (2022). 10.3390/ijms23073587 PMC8999085 35408946

[11] Brownlee, M. Biochemistry and molecular cell biology of diabetic complications. *Nature* **414** (6865), 813–820 (2001). 11742414 10.1038/414813a

[12] Dei Cas, A. et al. Impact of diabetes on epidemiology, treatment, and outcomes of patients with heart failure. *JACC Heart Fail.* **3** (2), 136–145 (2015). 25660838 10.1016/j.jchf.2014.08.004

[13] Capes, S. E., Hunt, D., Malmberg, K. & Gerstein, H. C. Stress hyperglycaemia and increased risk of death after myocardial infarction in patients with and without diabetes: a systematic overview. *Lancet* **355** (9206), 773–778 (2000). 10711923 10.1016/S0140-6736(99)08415-9

[14] Dungan, K. M., Braithwaite, S. S. & Preiser, J. C. Stress hyperglycaemia. *Lancet* **373** (9677), 1798–1807 (2009). 19465235 10.1016/S0140-6736(09)60553-5 PMC3144755

[15] Kosiborod, M. et al. Admission glucose and mortality in elderly patients hospitalized with acute myocardial infarction: implications for patients with and without recognized diabetes. *Circulation* **111** (23), 3078–3086 (2005). 15939812 10.1161/CIRCULATIONAHA.104.517839

[16] Stranders, I. et al. Admission blood glucose level as risk indicator of death after myocardial infarction in patients with and without diabetes mellitus. *Arch. Intern. Med.* **164** (9), 982–988 (2004). 15136307 10.1001/archinte.164.9.982

[17] Tang, Z. et al. Oxidative stress signaling mediated pathogenesis of diabetic cardiomyopathy. *Oxid Med Cell Longev* **2022**, 5913374 (2022). 35103095 10.1155/2022/5913374 PMC8800599

[18] Liu, J. & Hu, X. Association between glucose-to-lymphocyte ratio and in-hospital mortality in acute myocardial infarction patients. *PLoS One*. **18** (12), e0295602 (2023). 38060551 10.1371/journal.pone.0295602 PMC10703328

[19] Hu, T., Liu, X. & Liu, Y. Usefulness of glucose to lymphocyte ratio to predict in-Hospital mortality in patients with AECOPD admitted to the intensive care unit. *Copd* **19** (1), 158–165 (2022). 35392756 10.1080/15412555.2022.2052272

[20] Zhang, Y. & Zhang, S. Prognostic value of glucose-to-lymphocyte ratio in critically ill patients with acute respiratory distress syndrome: A retrospective cohort study. *J. Clin. Lab. Anal.* **36** (5), e24397 (2022). 35358348 10.1002/jcla.24397 PMC9102764

[21] Johnson, A. E. W. et al. MIMIC-IV, a freely accessible electronic health record dataset. *Sci. Data*. **10** (1), 1 (2023). 36596836 10.1038/s41597-022-01899-x PMC9810617

[22] Goldberger, A. L. et al. PhysioBank, physiotoolkit, and physionet: components of a new research resource for complex physiologic signals. *Circulation* **101** (23), E215–220 (2000). 10851218 10.1161/01.cir.101.23.e215

[23] Zhong, A., Cheng, C. S., Kai, J., Lu, R. & Guo, L. Clinical significance of glucose to lymphocyte ratio (GLR) as a prognostic marker for patients with pancreatic Cancer. *Front. Oncol.* **10**, 520330 (2020). 33117673 10.3389/fonc.2020.520330 PMC7561421

[24] Yang, M. et al. Glucose to lymphocyte ratio predicts prognoses in patients with colorectal cancer. *Asia Pac. J. Clin. Oncol.* **19** (4), 542–548 (2023). 36479824 10.1111/ajco.13904

[25] Liu, L. et al. Preoperative glucose-to-lymphocyte ratio predicts survival in cancer. *Front. Endocrinol. (Lausanne)*. **15**, 1284152 (2024). 38501103 10.3389/fendo.2024.1284152 PMC10946689

[26] Tang, S., Zhang, Z., Wang, Y. & Li, Y. Association between red blood cell distribution width-platelet ratio (RPR) and mortality in patients with heart failure from the MIMIC IV database: A retrospective cohort study. *Heliyon* **10** (16), e35796 (2024). 39247340 10.1016/j.heliyon.2024.e35796 PMC11378892

[27] Núñez, J. et al. Low lymphocyte count and cardiovascular diseases. *Curr. Med. Chem.* **18** (21), 3226–3233 (2011). 21671854 10.2174/092986711796391633

[28] Cioca, D. P., Watanabe, N. & Isobe, M. Apoptosis of peripheral blood lymphocytes is induced by catecholamines. *Jpn Heart J.* **41** (3), 385–398 (2000). 10987355 10.1536/jhj.41.385

[29] Westermann, J. & Bode, U. Distribution of activated T cells migrating through the body: a matter of life and death. *Immunol. Today*. **20** (7), 302–306 (1999). 10379047 10.1016/s0167-5699(99)01474-7

[30] Karwi, Q. G. et al. Weight loss enhances cardiac energy metabolism and function in heart failure associated with obesity. *Diabetes Obes. Metab.* **21** (8), 1944–1955 (2019). 31050157 10.1111/dom.13762

[31] Suskin, N. et al. Glucose and insulin abnormalities relate to functional capacity in patients with congestive heart failure. *Eur. Heart J.* **21** (16), 1368–1375 (2000). 10952826 10.1053/euhj.1999.2043

[32] Paolisso, G. et al. Prognostic importance of insulin-mediated glucose uptake in aged patients with congestive heart failure secondary to mitral and/or aortic valve disease. *Am. J. Cardiol.* **83** (9), 1338–1344 (1999). 10235092 10.1016/s0002-9149(99)00097-1

[33] Amato, L. et al. Congestive heart failure predicts the development of non-insulin-dependent diabetes mellitus in the elderly. The osservatorio geriatrico regione campania group. *Diabetes Metab.* **23** (3), 213–218 (1997). 9233998

[34] Ashrafian, H., Frenneaux, M. P. & Opie, L. H. Metabolic mechanisms in heart failure. *Circulation* **116** (4), 434–448 (2007). 17646594 10.1161/CIRCULATIONAHA.107.702795

[35] Lopaschuk, G. D., Ussher, J. R., Folmes, C. D., Jaswal, J. S. & Stanley, W. C. Myocardial fatty acid metabolism in health and disease. *Physiol. Rev.* **90** (1), 207–258 (2010). 20086077 10.1152/physrev.00015.2009

[36] Paolisso, G. et al. Advancing age and insulin resistance: role of plasma tumor necrosis factor-alpha. *Am. J. Physiol.* **275** (2), E294–299 (1998). 9688632 10.1152/ajpendo.1998.275.2.E294

[37] Geerlings, S. E. & Hoepelman, A. I. Immune dysfunction in patients with diabetes mellitus (DM). *FEMS Immunol. Med. Microbiol.* **26** (3–4), 259–265 (1999). 10575137 10.1111/j.1574-695X.1999.tb01397.x

[38] Marhoffer, W., Stein, M., Maeser, E. & Federlin, K. Impairment of polymorphonuclear leukocyte function and metabolic control of diabetes. *Diabetes Care*. **15** (2), 256–260 (1992). 1547682 10.2337/diacare.15.2.256

[39] Elenkov, I. J., Wilder, R. L., Chrousos, G. P. & Vizi, E. S. The sympathetic nerve–an integrative interface between two supersystems: the brain and the immune system. *Pharmacol. Rev.* **52** (4), 595–638 (2000). 11121511

[40] Lastra, G., Dhuper, S., Johnson, M. S. & Sowers, J. R. Salt, aldosterone, and insulin resistance: impact on the cardiovascular system. *Nat. Rev. Cardiol.* **7** (10), 577–584 (2010). 20697411 10.1038/nrcardio.2010.123

[41] Mann, D. L. Innate immunity and the failing heart: the cytokine hypothesis revisited. *Circ. Res.* **116** (7), 1254–1268 (2015). 25814686 10.1161/CIRCRESAHA.116.302317 PMC4380242

[42] Packer, M. The neurohormonal hypothesis: a theory to explain the mechanism of disease progression in heart failure. *J. Am. Coll. Cardiol.* **20** (1), 248–254 (1992). 1351488 10.1016/0735-1097(92)90167-l

