# Supplementary material for: Association between glucose-to-lymphocyte ratio and mortality in patients with heart failure from the MIMIC-IV database: a retrospective cohort study
Source: Sci Rep. 2025 Jul 1;15:21131. doi: 10.1038/s41598-025-08349-9 (PMC12219531; doi:10.1038/s41598-025-08349-9)
Supplement: Supplementary file 1 — Supplementary Material 1 [file 41598_2025_8349_MOESM1_ESM.docx]

Supplementary Material Table S1

| Variable | variance inflation factor (VIF) |
| --- | --- |
| Gender | 1.16 |
| Myocardial infarction | 1.08 |
| Cerebrovascular disease | 1.04 |
| Diabetes | 1.37 |
| Hypertension | 1.10 |
| AF | 1.16 |
| beta-blocker | 1.17 |
| ACEI/ARB/ARNI | 1.16 |
| Diuretic | 1.19 |
| Antidiabetic | 1.32 |
| Age | 1.47 |
| Hemoglobin | 3.03 |
| Platelet | 1.18 |
| WBC | 1.15 |
| Anion gap | 1.60 |
| BUN | 1.86 |
| Creatinine | 2.14 |
| Sodium | 1.07 |
| Potassium | 1.22 |
| Calcium | 1.12 |
| RBC | 2.58 |
| RDW | 1.30 |
| Weight | 1.29 |
| HR | 1.45 |
| SBP | 1.64 |
| DBP | 1.84 |
| RR | 1.25 |
| SpO2 | 1.15 |
| SOFA | 1.55 |
