# Supplementary material for: Association between glucose-to-lymphocyte ratio and mortality in patients with heart failure from the MIMIC-IV database: a retrospective cohort study
Source: Sci Rep. 2025 Jul 1;15:21131. doi: 10.1038/s41598-025-08349-9 (PMC12219531; doi:10.1038/s41598-025-08349-9)
Supplement: Supplementary file 2 — Supplementary Material 2 [file 41598_2025_8349_MOESM2_ESM.docx]

Table S2. Information of ROC curves in Figure 5.

| Model | AUC (95% CI) | Accuracy (95%CI) | Sensitivity (95%CI) | Specificity (95%CI) | PPV (95%CI) | NPV (95%CI) | Threshold |
| --- | --- | --- | --- | --- | --- | --- | --- |
| 30-day |  |  |  |  |  |  |  |
| GLR | 0.608 (0.60 - 0.62) | 0.53 (0.52-0.53) | 0.48 (0.48 - 0.49) | 0.68 (0.67 - 0.70) | 0.86 (0.85 - 0.87) | 0.25 (0.24 - 0.26) | 129.241 |
| Glucose | 0.555 (0.54 - 0.57) | 0.54 (0.53-0.54) | 0.53 (0.52 - 0.53) | 0.57 (0.55 - 0.59) | 0.83 (0.82 - 0.84) | 0.23 (0.22 - 0.24) | 154.5 |
| Lymphocyte | 0.596 (0.58 - 0.61) | 0.36 (0.35-0.37) | 0.32 (0.31 - 0.33) | 0.53 (0.51 - 0.54) | 0.73 (0.72 - 0.74) | 0.16 (0.16 - 0.17) | 0.883 |
| 365-day |  |  |  |  |  |  |  |
| GLR | 0.606 (0.60 - 0.62) | 0.58 (0.57-0.59) | 0.57 (0.56 - 0.58) | 0.60 (0.58 - 0.61) | 0.67 (0.66 - 0.68) | 0.49 (0.48 - 0.50) | 143.932 |
| Glucose | 0.534 (0.53 – 0.55) | 0.53 (0.52-0.54) | 0.50 (0.49 - 0.51) | 0.57 (0.56 - 0.58) | 0.63 (0.61 - 0.64) | 0.44 (0.43 - 0.45) | 149.5 |
| Lymphocyte | 0.607 (0.60 – 0.62) | 0.42 (0.41-0.43) | 0.43 (0.42 - 0.44) | 0.40 (0.39 - 0.41) | 0.51 (0.50 - 0.52) | 0.33 (0.32 - 0.34) | 1.145 |
